# Therapeutic Effect of Superficial Scalp Hypothermia on Chemotherapy-Induced Alopecia in Breast Cancer Survivors

**DOI:** 10.3390/jcm13185397

**Published:** 2024-09-12

**Authors:** Kefah Mokbel, Alevtina Kodresko, Jon Trembley, Hussam Jouhara

**Affiliations:** 1 The London Breast Institute, Princess Grace Hospital, London W1U 5NY, UK; 2 Heat Pipe and Thermal Management Research Group, College of Engineering, Design and Physical Sciences, Brunel University, London UB8 3PH, UK; 3 Air Products PLC, Hersham Place Technology Park, Molesey Road, Surrey KT12 4RZ, UK; trembljp@airproducts.com; 4 Vytautas Magnus University, Studentu Street 11, Kaunas District, LT-53362 Akademija, Lithuania

**Keywords:** alopecia, breast cancer, chemotherapy-induced alopecia, cryotherapy, hair loss, scalp hypothermia, scalp cooling

## Abstract

Alopecia is a common adverse effect of neoadjuvant or adjuvant chemotherapy in patients with early breast cancer. While hair typically regrows over time, more than 40% of patients continue to suffer from permanent partial alopecia, significantly affecting body image, psychological well-being, and quality of life. This concern is a recognized reason why some breast cancer patients decline life-saving chemotherapy. It is critical for healthcare professionals to consider the impact of this distressing side effect and adopt supportive measures to mitigate it. Among the various strategies investigated to reduce chemotherapy-induced alopecia (CIA), scalp cooling has emerged as the most effective. This article reviews the pathophysiology of CIA and examines the efficacy of different scalp cooling methods. Scalp cooling has been shown to reduce the incidence of CIA, defined as less than 50% hair loss, by 50% in patients receiving chemotherapy. It is associated with high patient satisfaction and does not significantly increase the risk of scalp metastasis or compromise overall survival. Promising new scalp cooling technologies, such as cryogenic nitrogen oxide cryotherapy, offer the potential to achieve and maintain lower scalp temperatures, potentially enhancing therapeutic effects. Further investigation into these approaches is warranted. Research on CIA is hindered by significant heterogeneity and the lack of standardised methods for assessing hair loss. To advance the field, further interdisciplinary research is crucial to develop preclinical models of CIA, establish a uniform, internationally accepted and standardised classification system, and establish an objective, personalised prognosis monitoring system.

## 1. Body Image in Women Diagnosed with Breast Cancer

Over the past several decades, early detection and advances in breast cancer therapies have revolutionized its management. Despite the promising survival rates, novel systemic anti-cancer therapies have also introduced a variety of dermatological toxicities, significantly increasing the number of patients living with these sequelae [1]. The most commonly used agents for breast cancer generally target rapidly dividing cells and are therefore associated with a myriad of mucocutaneous toxicities that affect hair, skin, nails, and mucosal surfaces due to their high turnover rate of epithelial cells. The most commonly encountered chemotherapy-induced dermatological toxicities include alopecia, hand–foot syndrome, mucositis, and nail changes, where alopecia consistently ranks among the most traumatic and troublesome aspects and can lead to functional impairment and physical and emotional distress. Between 40% and 100% of breast cancer patients experience complete alopecia after cytotoxic chemotherapy [2]. The state of a person’s scalp is regarded as a gender-identity marker and provides psychosocial signals that range from general well-being to social status across most cultures globally. Although hair regrows after chemotherapy, over 40% of patients still experience permanent partial alopecia three years later [2].

Research has shown that changes such as chemotherapy-induced alopecia can significantly affect breast cancer survivors’ valuation of their bodies, stimulating the development of unhealthy behavioural patterns and spirals of negative emotions and therefore impairing quality of life and altering self-image, cultural identity, sexuality, femininity, and mental health. The alopecia stigmata of breast cancer therapy can be so potent that patients may develop anxiety, depression, social withdrawal, fundamental identity changes, and body dysmorphic disorder, or may even decline further therapy. Furthermore, recent research suggests that some women with breast cancer have reported that chemotherapy-induced alopecia is even more difficult to cope with than mastectomy. On top of that, the negative repercussions of chronic psycho emotional stress related to chemotherapy-induced alopecia can compromise anti-tumour immune defences and recovery from hair loss. According to animal models, associated stress mediators can induce severe neurogenic inflammation, inhibit the growth of human-scalp hair follicles, and promote telogen effluvium [3]. In accordance with body image theories and integrative reviews of the physical, psychological, and social experiences of breast cancer survivors who have experienced chemotherapy-induced alopecia, body image has been identified as a vital factor directly related to psychological and physical wellbeing both post-chemotherapy and throughout the cancer journey [4,5]. In this context, recognition and management of body image as a dynamic and multidimensional phenomenon and associated body acceptance concerns demand increased attention, given the poignant and lasting effects [6].

### Chemotherapy-Induced Alopecia in Breast Cancer Survivors and Current Unmet Needs

**Figure 1:**
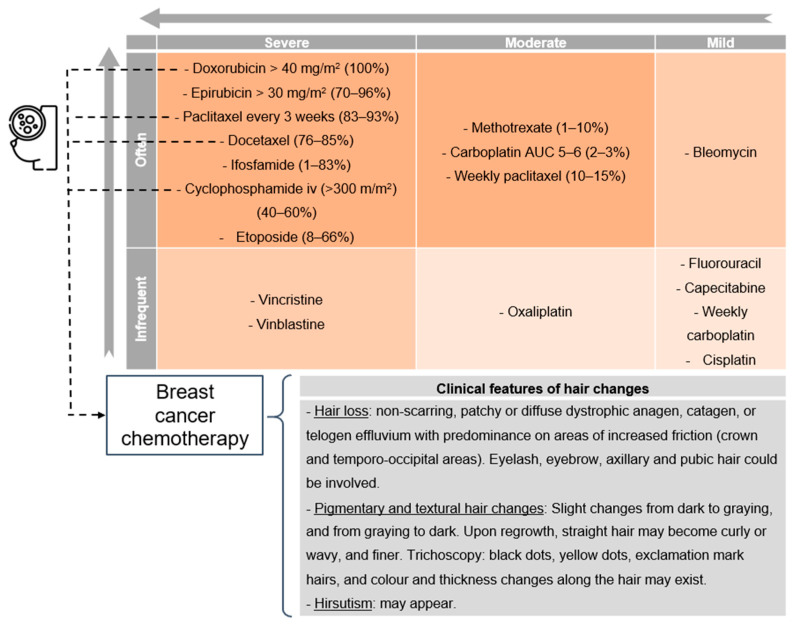
Chemotherapy agents, risk of alopecia, and their application in breast cancer therapies and associated hair toxicity.

Chemotherapy-induced alopecia is the most common and visually striking toxicity, with its incidence and severity dependent on the class, route, dose, and schedule of the cytotoxic drugs used and the response of the individual [1]. As shown in Figure 1, certain classes of chemotherapy agents are known to cause alopecia more readily, with an incidence of up to 60–100% with topoisomerase inhibitors (e.g., doxorubicin), 80% with antimicrotubule agents (e.g., paclitaxel), >60% with alkylating agents (e.g., cyclophosphamide), and 10–50% with antimetabolites. These are associated with the most severe and frequent alopecia consequences and are the most common in chemotherapy for breast cancer.

**Figure 2:**
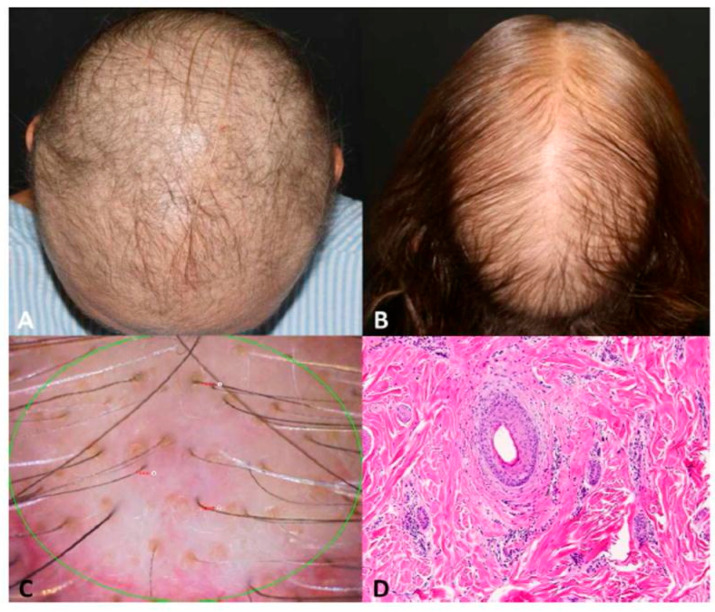
Persistent chemotherapy-induced alopecia in breast cancer survivors: (**A**) diffuse alopecia 2 years after taxane-based chemotherapy completion; (**B**) persistent chemotherapy-induced alopecia 1.6 years after taxane-based chemotherapy completion with similar pattern of androgenetic alopecia and predominant hair thinning on the crown area; (**C**) trichoscopy of patient in (**B**) with miniaturized hairs, showing hair thinning and yellow dots. (**D**) Histology section showing fibrosis and mild perifollicular inflammation (hematoxylin–eosin stain) [9].

Clinically, chemotherapy-induced alopecia manifests as diffuse or patchy, depending on the distribution of hairs in the active anagen phase and nonscarring alopecia that begins within 2 weeks of the onset of chemotherapy and is often well established by 2 to 3 months. It is usually categorized as diffuse anagen or telogen effluvium, depending on the hair follicle response to the same damage that is capable of stopping mitosis with both shedding patterns. The pattern of hair loss seems to be lost first from the crown and sides of the head above the ears. In the literature, it is believed that most cases resolve with gradual hair regrowth 3–6 months after chemotherapy is discontinued. Published clinical experience and reports reveal that new hair strands can be of a different colour, thickness, texture, and waviness. Although usually reversible, cases of chemotherapy-induced permanent or irreversible alopecia have been reported and can be particularly devastating [7]. The extent of damage to epithelial hair-follicle stem cells seems to play a crucial role. In recent reviews of the literature, lack of or incomplete hair regrowth 6 months after chemotherapy cessation has been observed after treatment with adjuvant chemotherapy with taxanes for breast cancer, as shown in Figure 2. In a study of breast cancer, permanent alopecia was reported by 23.3% of patients receiving docetaxel and 10.1% receiving paclitaxel [8]. Another study reported permanent alopecia in 39.5% and 42.3% at 6 months and 3 years, respectively.

Despite the increasing incidence of chemotherapy-induced alopecia in cancer survivors, particularly breast cancer, and given its profound impact on the quality of life, many oncology centres still do not routinely offer services for the management of alopecia except for wigs. While proper counselling prior to hair loss softens its impact, guidelines or recommendations specifically for the prevention and treatment are lacking, primarily due to the limited understanding of the human chemotherapy-induced alopecia mechanisms, predictive experimental models, and therefore effective treatment and therapeutic approaches [10]. In the current era of patient-centered care, both clinicians and researchers must endeavour to address this dermatological toxicity by establishing interventions that can ameliorate the distress of this burdensome event for millions of cancer survivors who receive chemotherapy across the globe each year [11].

## 2. Chemotherapy and Hair Loss: Underlying Principles of Pathobiology

### 2.1. Hair Follicle Biology

**Figure 3:**
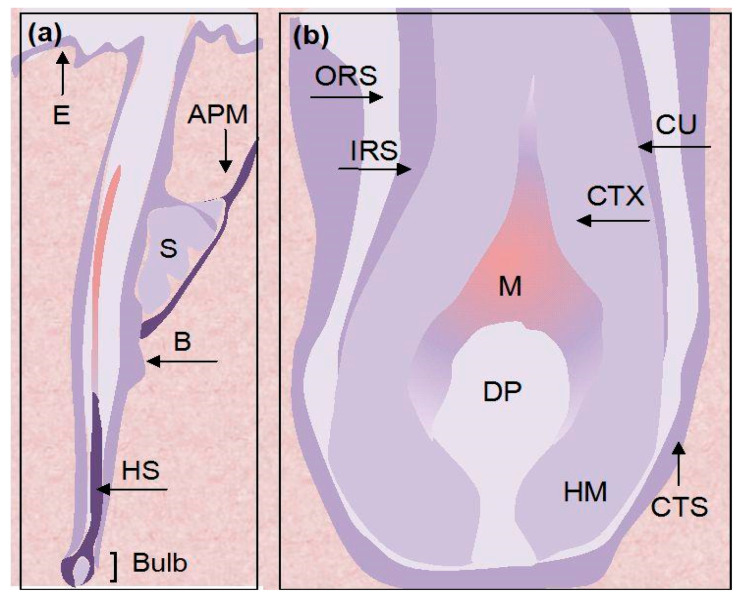
Schematic representation of structure of hair follicle in its mature anagen phase: (**a**) a full-length longitudinal view of hair follicle; (**b**) hair follicle bulb. Abbreviations: APM, arector pili muscle; B, bulge; CTS, connective tissue sheath; CTX, cortex of hair shaft; CU, cuticle of hair shaft; DP, dermal papilla; E, epidermis; HM, hair matrix; HS, hair shaft; IRS, inner root sheath; M, melanocytes; ORS, outer root sheath; S, sebaceous gland [12].

Hair is a derivative of the epidermis, where it sits in the hair follicle. In human beings, all hair follicle formation is complete at birth, and of the total number of 5 million hair follicles, 100,000 are located on the scalp. Each strand of hair comprises the following two separate structures: a hair shaft, which is a thin, flexible cylinder of non-living, keratinized epithelial cells above the epidermis, and a living hair follicle that lies underneath the epidermis while being embedded in the connective tissue and subcutaneous fat. The hair follicle, shown in Figure 3, is a highly organized and essential growth structure of the hair that has the following two distinct parts: the upper part, consisting of the isthmus and infundibulum, and the lower part, made of the hair bulb and suprabulbar region. The expanded onion-shaped portion—the hair bulb that encloses the hair matrix and the follicular papilla—is known as the active reproductive portion of the hair follicle [12]. 

The papilla at the base of the bulb is believed to be a primary orchestrator and an essential source of growth factors in the hair growth process that provides vital blood supply to the growing hair follicle. The dermal sheath surrounding the dermal papilla is composed of progenitor cells that possess wound-healing properties, rejuvenate the dermal papilla, and regulate the hair cycle. Matrix cells are other unspecialized epithelial cells that surround the dermal papilla at its bulge region and act as germ cells that proliferate and function to grow the hair follicle and, subsequently, the hair by forming an outwardly moving hair shaft from the dermis. The continuous division of matrix cells also eventually differentiates into the internal root sheath and keratin-producing cells. Melanocytes residing among matrix stem cells are responsible for the pigmentation of hair by transferring the melanin granule to the keratinocytes of the growing hair shaft. Keratinization is completed as the cells are pushed to the skin surface to form the shaft of differentiated hair, which consists of a medullary core surrounded by a cortex and cuticle. Extensive research in anatomical and physiological aspects of hair demonstrated that damage to the dermal papilla is associated with non-scarring alopecia, while irreversible alopecia is a result of damage to the matrix of the hair follicle bulge that contains epithelial stem cells [13].

**Figure 4:**
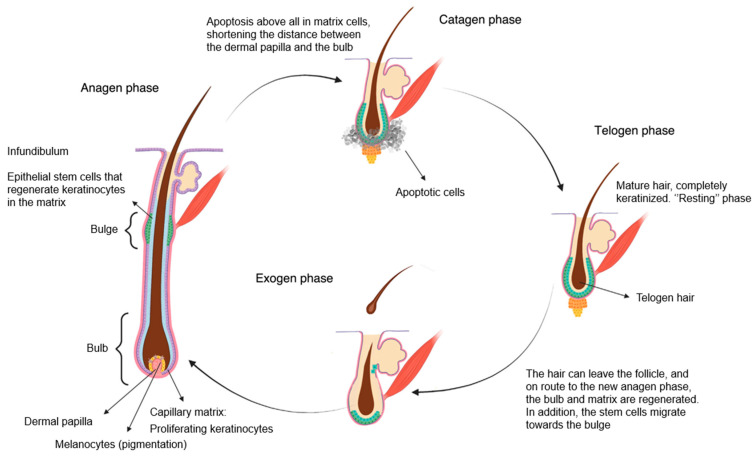
Normal cycle of hair growth [13].

Once formed, the hair follicles undergo life cycles unique to the hair follicle and not observed in any other mammalian structure. These continuous rhythmic changes driven by apoptotic signals are characterized by stages of rapid growth and elongation of the hair shaft that alternate with periods of quiescence and regression. Figure 4 depicts phases of the normal cycle of hair growth that can be divided into the following three phases: anagen, catagen, and telogen, after which shedding, or exogen, occurs. Between 80% and 90% of hairs are in the anagen phase, which lasts for a period of 2–6 years during which mitotically active cells of the matrix found in the hair bulb differentiate and divide, resulting in the actual production of hair fiber. Next is catagen, the transition phase, where growth stops, and the follicle undergoes apoptosis-driven regression. After that, the hair enters the telogen phase, also known as the resting phase, when all mitotic activity stops, and the fully keratinized hair is ready to be shed. Although the exact mechanism that controls phase progress is not fully explored, the bulge activation theory postulates that the growth-phase transitions eventuate when the bulge stem cells are activated by growth factors produced in the dermal papilla. The transient amplifying nature of these cells allows them to only go through a limited number of mitoses, which sets the duration of anagen and the onset of catagen phases.

### 2.2. Pathophysiology

**Figure 5:**
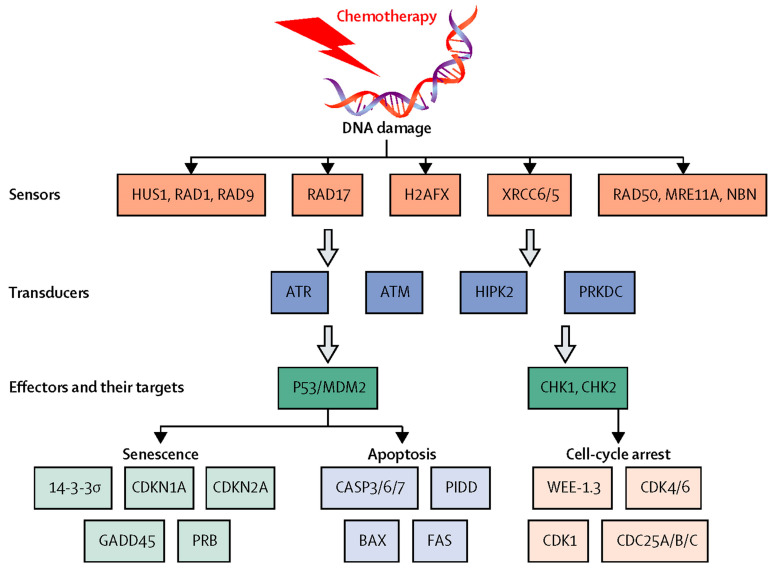
Molecular damage-response pathways activated by chemotherapy [3].

The term alopecia refers to the partial or total absence of hair in any area where it is expected to be present. Alopecia has many variants that are categorized as localized or diffuse, temporary or permanent, and scarring or non-scarring. Chemotherapy-induced alopecia is most prominent on the scalp, presents suddenly, and initially manifests as patterned hair loss with a predilection for areas with low total hair density, such as the frontal or occipital hairlines. Hair-shaft shedding manifests as diffuse anagen or telogen effluvium and results from defined disturbances of normal hair-shaft production and of hair-follicle cycling. Its reversibility depends on the degree of damage to hair follicle stem cells. Although chemotherapeutic agents have distinct mechanisms of action and hair loss patterns, the overall final clinical presentation of chemotherapy-induced alopecia across patients and the principal hair follicle damage-response pathways are similar [3]. The pathophysiology of chemotherapy-induced alopecia is complex and not fully understood. Much of the current understanding has been gleaned from research on an adult rat model, newborn rats, and the C57BL/6 mouse model. Many chemotherapeutic agents share proapoptotic pathways, especially p53-mediated signalling, which specifically plays a crucial role in chemotherapy-induced alopecia. Figure 5 illustrates molecular damage-response pathways that result in death by apoptosis in cells that are in the process of mitotic division, including the keratinocytes proliferating in the hair bulb.

**Figure 6:**
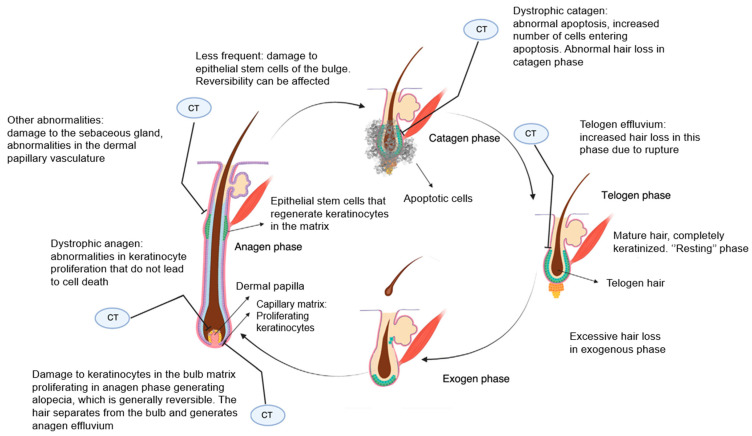
Main sites where chemotherapy affects the hair cycle and generates alopecia. CT, chemotherapy [13].

This causes alopecia through various mechanisms illustrated in Figure 6. In cases where keratinocyte proliferation is severely inhibited, the hair will separate in the bulb and shed, with an abrupt loss of hair in the growth phase, in a process known as anagen effluvium. If dystrophic anagen effluvium happens, alopecia is less severe, and there is essentially a delay in hair growth due to delayed proliferation of keratinocytes. The affected structure and texture of the shafts are frequently of decreased quality and show pigmentary defects. Next, abnormal apoptosis can initiate more severe damage via the dystrophic catagen response pathway but paradoxically result in faster hair follicle recovery and regrowing hair shafts that retain a normal structure and are fully pigmented. Another mechanism is known as telogen effluvium, when breakage of the hair stem at the follicular orifice occurs during the telogen phase.

## 3. Cryotherapy and Scalp Hypothermia: Mode of Action and Its Emerging Role in Alopecia

Given the varied individuals’ inherent susceptibility and alopecia-inducing mechanisms of chemotherapies, chemotherapy-induced alopecia is a daunting therapeutic challenge. Although a number of different strategies have been assessed to manage hair follicle damage caused by chemotherapy, including pharmacological interventions such as topical minoxidil, finasteride, spironolactone, and topical calcitriol, to date, none of these have shown satisfactory results or been approved by regulatory agencies. Importantly, safety concerns have been raised due to oestrogen elevations in many cases. Accordingly, in the absence of pharmacological strategies, the management of alopecia in cancer survivors has been explored through various therapeutic approaches. To date, only one approach, scalp hypothermia, has been cleared by the US Food and Drug Administration (FDA) in 2015 for breast cancer survivors to prevent chemotherapy-induced alopecia [9,14,15].

**Figure 7:**
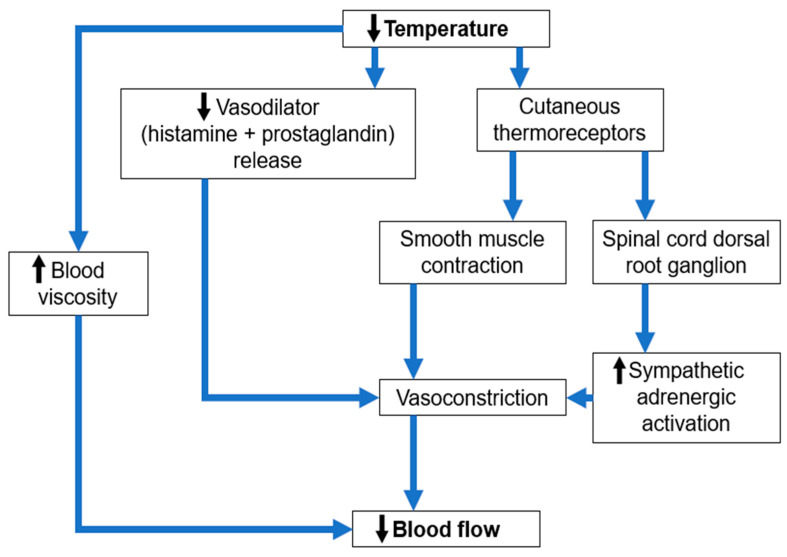
The mechanism cryotherapy affects blood flow.

Since the late 1970s, the therapeutic efficacy of superficial cryotherapy in alopecia has occupied a very important position in the armamentarium of dermatologists and has been advocated as a safe, effective, reproducible, repeatable, and simple approach. It is clearly evidenced that exposure of the human body to ultra-low temperatures results in many favourable physiological phenomena represented by the anti-inflammatory and analgesic effects following changes in the circulatory, immunological, neuromuscular, and endocrine systems. Primarily, this is achieved by cold stress, which initiates a biphasic vascular response that results in a mechanism of vasoconstriction and decreased blood flow, as summarized in Figure 7.

**Figure 8:**
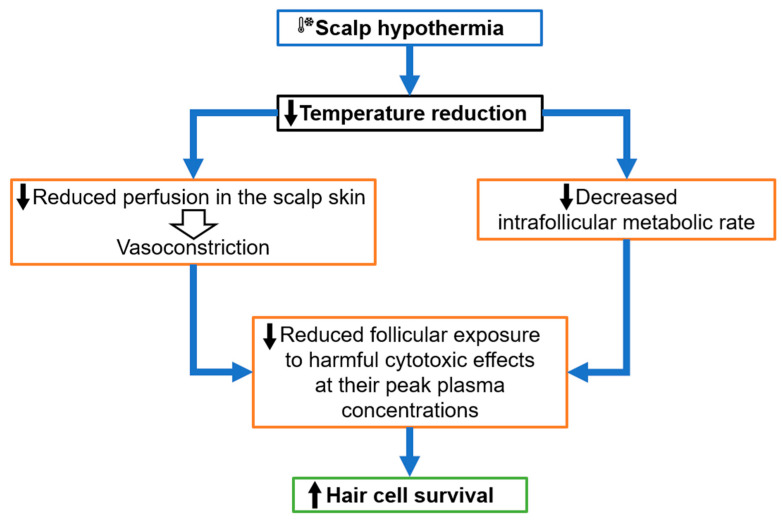
The beneficial effects of superficial scalp hypothermia in chemotherapy-induced alopecia.

In scalps exposed to chemotherapy, delivery of scalp hypothermia is theorized to act by causing vasoconstriction of blood vessels, reducing the amount of cytostatic and cytotoxic agents reaching the scalp, decreasing the intrafollicular metabolic rate, and follicular drug uptake and overall resulting in reduced follicular exposure to harmful cytotoxic effects at their peak plasma concentrations, as shown in Figure 8 [16]. Furthermore, induced cell cycle arrest at the G0/G1 phase, increased HSP70 accumulation for cell stress protection, and reduced cell apoptosis have also been proposed as potential mechanisms of protection against chemotherapy-induced alopecia [8].

In additional studies on other alopecias, such as areata, liquid nitrogen cryotherapy using a dip-stick or spray method is thought to induce hair regrowth through several mechanisms that include vasoconstriction and reactive vasodilatation, improving the microcirculation; partial damage or denaturation of keratinocytes decreasing the perifollicular cellular infiltrates; alteration of immunological processes further decreasing the T cell infiltration; reduced granzyme B production; reduced in vitro and in vivo T-cell and monocyte activation response; reduced IL-17 release in T cells; reduced IL-1β/IL-23 activation of T cells; blocking a pathology associated with abnormal melanocytes; and causing melanocyte alteration and preventing their role in the initiation of alopecia [17,18,19,20,21,22,23,24]. 

**Figure 9:**
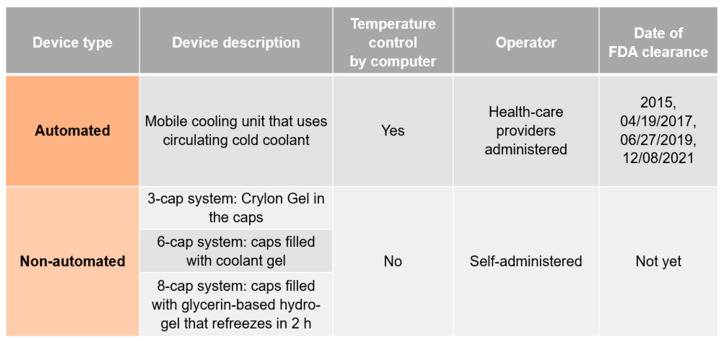
Scalp cooling devices used for chemotherapy-induced alopecia by patients with solid tumours.

**Figure 10:**
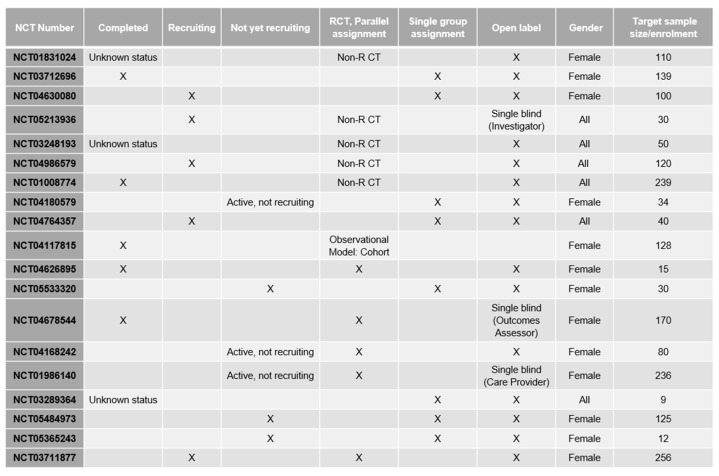
Current clinical trials [8].

Presently, two types of scalp cooling devices for patients with solid tumours undergoing chemotherapy regimens are available: automated and non-automated. Figure 9 and Figure 10 summarize the current evidence and current clinical trials on both FDA-cleared scalp cooling systems and self-administered cold caps, not cleared by the FDA, that are also available from several manufacturers.

**Figure 11:**
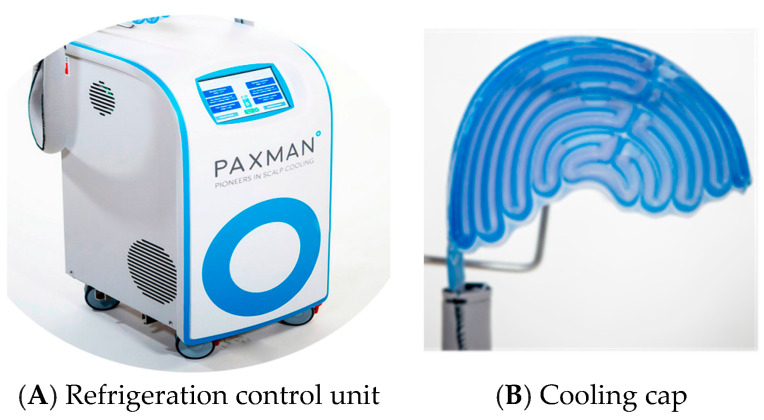
Automated scalp cooling system: a refrigeration unit (**A**) and a cooling cap (**B**) [25].

The approved automatic scalp cooling systems are computerized mobile units that circulate a refrigerant to maintain the scalp temperature within a narrow range. An example of such a system is demonstrated in Figure 11. The scalp temperature is maintained at 3–5 °C throughout chemotherapy and for 90 min to 120 min afterward, resulting in a subcutaneous (1–2 mm) scalp temperature of ~18–22 °C. Regardless of the device used, scalp cooling begins 30 min before chemotherapy, continues during the infusion, and after the conclusion of treatment. Although there is little evidence on the efficacy of non-automatic devices, a considerable number of studies on automatic systems have established a mean success rate of 50–70%.

## 4. Therapeutic Effect of Scalp Hypothermia in Breast Cancer: Current Evidence

**Figure 12:**
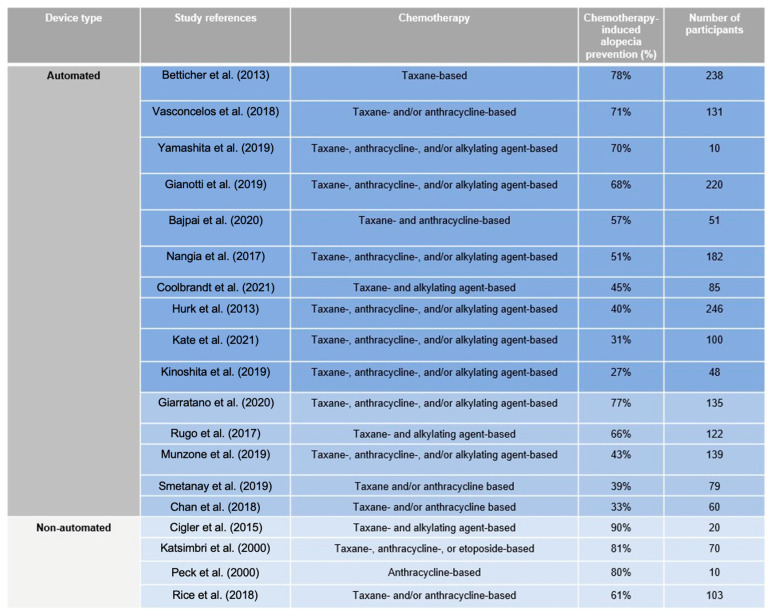
Current evidence on scalp cooling in chemotherapy-induced alopecia in breast cancer survivors [28,29,30,31,32,33,34,35,36,37,38,39,40,41,42,43,44,45,46].

As the field continues to expand and evolve, the last few years have witnessed a growth in supportive studies with promising results on scalp hypothermia in breast cancer survivors. Evidently, the effectiveness of scalp hypothermia for hair preservation depends on the device, with variable degrees and durations of cooling, chemotherapy agent and regimen, patient performance status, intrinsic patient characteristics, metabolism, hair thickness, head shape, and cap fitting [26]. Figure 12 summarizes the majority of the most recent studies. In one of the most recent meta-analyses encompassing 2179 chemotherapy patients, 60.7% experienced <50% hair loss. In 28 studies focused on taxane-based chemotherapy, the rate was 60.0%. Hair loss was significantly lower with scalp hypothermia (49.3%) compared to no treatment (0%). Scalp cooling resulted in less than 50% hair loss in paclitaxel- and docetaxel-based treatments. Patient satisfaction with scalp cooling was 78.9% [27].

### 4.1. Paxman Scalp Cooling System

Notably, the most researched cooling system for chemotherapy-induced alopecia in breast cancer survivors is the Paxman Scalp Cooling System. According to the wealth of literature on the topic, it is evident that the use of this device can significantly prevent alopecia with acceptable safety and result in faster recovery of hair volume. Evidence from a prospective observational single-centre study by Vasconcelos et al. [28] on breast cancer survivors receiving anthracycline/taxane-based and taxane-monotherapy-based chemotherapy has shown successful hair preservation in 71% of cases. Importantly, the success rate was significantly different among the chemotherapy regimens, with the highest success rates among patients receiving taxane-monotherapy-based therapy. The evidence was concordant with the findings of other reports, such as the study by Gianotti et al. [29] examining taxane- and/or anthracycline-based chemotherapy. The overall success rate was 68%, and severe hair loss was avoided in 89% of patients receiving taxane-based chemotherapy and in 78% receiving both anthracyclines and taxanes. Similarly, the study by Nangia et al. [30] on taxane-, anthracycline-, and/or alkylating agent-based chemotherapy revealed scalp cooling by Paxman to improve hair loss in 51% of patients. The evaluation of alopecia was grade 0 in 70% of patients after taxane-, anthracycline-, and/or alkylating agent-based chemotherapy in the study by Yamashita et al. [31]. Supportive findings were reported by Bajpai et al. [32], who examined breast cancer survivors receiving taxane- and anthracycline-based chemotherapy. The overall success rate of hair preservation was 57%, with significant differences in success depending on drug sequence. In patients who received taxanes first, 77% of patients had successful hair preservation, while in those who received anthracyclines first, only 33% had successful hair preservation. In a taxane-based chemotherapy-only study by Betticher et al. [33] on various tumours, including breast, lung, and prostate, cold caps and the Paxman Scalp Cooling System substantially reduced alopecia by 78%. In another taxane-based study by Coolbrandt et al. [34], scalp cooling was successful in preventing severe alopecia in 45% of breast cancer patients. In an observational study by Hurk et al. [35] on taxane-, anthracycline-, and/or alkylating agent-based chemotherapy, scalp cooling reduced the use of wigs and head covers by 40%. Kate et al. [36] observed scalp cooling to be more effective in reducing chemotherapy-induced alopecia in patients treated with taxane-based chemotherapy over anthracycline-based chemotherapy, and overall, grade 0–1 alopecia was experienced in 31% of the treated patients. In the study by Kinoshita et al. [37], the use of a scalp-cooling device prevented alopecia with acceptable safety in 27%, and an increase in hair volume of ≥50% within 12 weeks duration after chemotherapy was observed in 85.7% of scalp-cooled patients.

### 4.2. DigniCap Scalp Cooling System

Another remarkable example of scalp cooling used in chemotherapy-induced alopecia is the FDA cleared DigniCap Scalp Cooling System. Supportive evidence was demonstrated in a recent study by Giarratano et al. [38] in early breast cancer patients receiving taxane-, anthracycline-, and/or alkylating agent-based chemotherapy. The final success rate in alopecia prevention was 77% at 3 weeks from the start of chemotherapy and 60% at 3 weeks from the end of hypothermia application. Scalp cooling was well tolerated, and higher success rates were reported in non-anthracycline-containing regimens (71%) compared to anthracycline-containing regimens (54%), suggesting its specific efficiency for patients undergoing a taxane-based non-anthracycline regimen. Additionally, differences were found within the taxane-based therapy group, and weekly paclitaxel showed 100% prevention compared to 43% in three-weekly docetaxel plus cyclophosphamide. Interestingly, the role of chemotherapy sequence on the scalp cooling success rate was observed, with a higher final success rate obtained in patients who underwent anthracyclines after taxanes than in those who underwent the same chemotherapy sequence but in reverse order. A theoretical biological explanation could be that the weekly administration of taxanes permitted sustained scalp hypothermia as well as decreased follicular metabolism and the subsequent delivery of anthracyclines, limiting the anthracycline-related follicular damage. These results are consistent with those from a multi-center cohort study by Rugo et al. [39], who evaluated taxane- and alkylating agent-based chemotherapy and observed better hair preservation in the paclitaxel-alone group (83%) compared to the docetaxel plus cyclophosphamide group (61%). Scalp cooling allowed hair loss of 50% or less to be seen in 66% of early-stage breast cancer survivors, overall highlighting its potential to lower the risk of alopecia after non-anthracycline-based chemotherapy. The study by Chan et al. [40] on taxane- and/or anthracycline-based chemotherapy reported minimal hair loss as a result of scalp cooling therapy in 33% of cases but most importantly supported the claim that patients receiving taxane-only chemotherapy would benefit from the therapy the most, while those receiving a combination of anthracycline and taxane chemotherapy tend to have the lowest rates of minimal hair loss. Evidence from Smetanay et al. [41] on taxane and/or anthracycline-based chemotherapy demonstrated supportive scalp cooling efficiency with hair preservation observed in 39% of patients. On the other hand, no differences were observed in the efficacy or quality of life between anthracycline- and taxane-based regimens. In a prospective study by Munzone et al. [42] on breast cancer patients who received taxane-, anthracycline-, and/or alkylating agent-based treatment, but mainly anthracycline-containing adjuvant treatment, scalp cooling safely prevented hair loss in 43%.

### 4.3. Penguin Cold Caps

In a plethora of research, there is also a growing body of literature that substantiates initial but robust evidence on the efficiency of cold cap systems such as Penguin Cold Caps in preventing chemotherapy-induced alopecia among patients with breast cancer. In a prospective study by Cigler et al. [43], excellent or good hair preservation was maintained in 90% of women with early-stage breast cancer receiving adjuvant docetaxel and cyclophosphamide chemotherapy (taxane- and alkylating agent-based). Scalp cooling was well tolerated, with no patient discontinuing therapy because of an intolerance to cold caps. In the study by Rice et al. [44], scalp hypothermia with the Penguin Cold Caps successfully prevented alopecia in 61% of patients treated with taxane- and/or anthracycline-based chemotherapy, but efficacy was chemotherapy regimen-specific. The system was the most effective for non-anthracycline-based shorter regimens, with rates of 100% for docetaxel-carboplatin with or without trastuzumab, or 84% for docetaxel-cyclophosphamide. A study by Katsimbri et al. [45] also evaluated the Penguin Cold Caps in patients with various main tumour types, including breast cancer (13%), that received taxane-, anthracycline-, or etoposide-based chemotherapy. The system was well tolerated, and an overall 81% hair loss protection rate was achieved. Patients receiving anthracyclines or etoposides had absolute protection, and the system demonstrated effective protection from alopecia caused by taxanes in 88% of patients, with 41% having absolute protection. Importantly, encouraging protection has been shown in 36% of patients treated with the combination of taxanes and anthracyclines since the synergism is associated with significant alopecia without protection. A small pilot study conducted by Peck et al. [46] similarly indicated beneficial effects in women receiving anthracycline-based chemotherapy for breast cancer, namely multiple cycles of the FEC regimen that includes fluorouracil, epirubicin, and cyclophosphamide. Overall, 70% of patients did not require a wig at the end of treatment, and 10% of patients withdrew at cycle 2, but at this point had experienced only minor hair loss and did not require a wig. 

### 4.4. Oncological Safety

Scalp cooling appears to be ontologically safe in relation to scalp metastasis. Rugo et al. [47] reviewed 24 studies, with 10 quantifying scalp metastasis incidence over time. Among 1959 patients using scalp cooling, the incidence was 0.61%, compared to 0.41% in 1238 patients without scalp cooling. The incidence of scalp metastases was low in both groups, indicating that scalp cooling does not increase the risk [47].

However, the data are limited in relation to systemic recurrence and overall survival. In a retrospective cohort study of 1370 women with non-metastatic invasive breast carcinoma who received neoadjuvant or adjuvant chemotherapy, scalp cooling to prevent chemotherapy-induced alopecia had no negative impact on survival [48].

While scalp cooling reduces hair loss in early-stage breast cancer patients, it may also protect circulating tumour cells (CTCs) in the scalp from chemotherapy by reducing blood flow through vasoconstriction and metabolic rate through inducing cell cycle growth G0/G1 phase arrest. These protected CTCs could potentially disseminate to other metastatic sites. Therefore, additional long-term survival data are needed to confirm the oncological safety of scalp cooling, especially in high-risk patients.

### 4.5. Challenges and Future Perspectives

In accordance with the present studies, it is evident that the efficiency of scalp cooling varies with the type of device and chemotherapy regimen. The consensus view seems to support the claim that scalp cooling is more effective for patients receiving taxane-based chemotherapy, including docetaxel or paclitaxel, compared with anthracycline-based chemotherapy, which requires an individualised approach, balancing the low rates of efficacy and longer treatment times against the importance of hair loss perceived by the patient. In the matter of permanent or irreversible alopecia, conclusions are yet to be drawn due to the lack of data.

Current research appears to validate the safety of scalp hypothermia, which may result only in mild adverse events, usually including discomfort for the patient, and it does not increase the risk of cutaneous scalp metastases, nor does it compromise cancer outcomes. The automated scalp cooling systems are cleared by the FDA for the expanded use of scalp cooling systems for patients with solid tumours undergoing chemotherapeutic protocols associated with a high risk of developing chemotherapy-induced alopecia. It is recommended in the National Comprehensive Cancer Network guidelines with a level of evidence IIA and with a level of evidence IIB in the new guidelines for management of adverse effects issued by the European Society for Medical Oncology. Nevertheless, scalp cooling is not applicable to all chemotherapy regimens, such as those involving platinum derivatives associated with the development of severe peripheral neuropathies, and should be avoided in patients with cryoglobulinemia, cold agglutinin disease, post-traumatic dystrophy caused by cold and cold-induced migraines, as well as in patients with haematologic malignancies, central nervous system malignancies, skin, head, and neck cancers, and in those who undergo bone marrow or stem cell transplantation with myelosuppressive doses of chemotherapy and/or radiotherapy.

Notwithstanding the preliminary robust efficiency of these cooling systems, several concerns can be pointed out from the studies, hindering the new potential findings and insights and, therefore, the widespread application of scalp cooling. Notably, the major limitations of these studies are the variability of study design, empirical patient selection, small sample sizes, proportions of patients in groups, absence of a control arm, lack of randomization and blinding, observational nature of studies, results being based on retrospective reports, and monocentric evaluations. Accordingly, these factors may contribute to discrepancies in success rates and limit the comparison of hair preservation rates between different studies. Furthermore, it could be one of the factors of reported inconsistency in improvements in patients’ quality of life. 

Another important aspect that should be acknowledged is the achievement and maintenance of optimal subcutaneous scalp temperature, which greatly varies among studies. Currently, there is no evidence for a cut-off point at which hair loss can be prevented by scalp hypothermia. Although research has shown that a subcutaneous temperature of 22 °C has to be maintained for 20 min before significant protection can be achieved, other pre-clinical studies have demonstrated that greater effects in prevention could be attained with 15 °C. In view of the efficacy of scalp cooling being reliant upon scalp temperature, the possibility of achieving and maintaining lower scalp temperatures should be investigated, and scalp cooling times should be additionally established. Specifically, owing to the growing body of evidence from studies on the efficiency of liquid nitrogen cryotherapy using a dip-stick or spray method in alopecia areata, it is of interest and has great potential to examine cryogenic cryotherapy to manage chemotherapy-induced alopecia while maintaining patient safety. Liquid nitrogen cryotherapy proved to be effective and safe in alopecia areata, with 75% of the patients having repopulated the alopecic plaques in response to treatment, while the cotton-tipped applicator method achieved >90% growth in 62.5% of patients, 75% growth in 25%, 50% in 5%, and no hair growth in 7.5%. Other advantages included safety, ease of use, low cost, and the capability to treat small children. 

The next important and challenging aspect is the absence of an optimal outcome measure for the severity of chemotherapy-induced alopecia, and the endpoints differ considerably between the studies. These subjective standardized or non-standardized assessments based on patient self-evaluation by the clinician, which include WHO criteria, the VAS scale, head cover use, the Common Toxicity Criteria and Dean’s scale, have the potential to contain cognitive bias and complicate any direct comparisons and therefore the definitive evaluation of efficacy between studies. Another concern is the cost and practical difficulties of adopting scalp cooling, including the variability of the proper fit of the cap, which is key to successful hair retention. This could have led to the scarcity of research and a high drop-out rate in some studies.

Good management of the device by the nursing team and medical staff commitment are the fundamental factors in providing support and motivation to bear the discomfort and mild-related adverse events, as well as the implementation of scalp cooling in the daily practice of oncology units. In this context, significant advances will only be made if adequate funding and interdisciplinary research efforts are invested, firstly into the development of preclinical models of chemotherapy-induced alopecia, a uniform, internationally standardized method for its classification, and an objective, individualised prognosis system. 

The development of validated objective quality of life tools and outcome measures for the severity of chemotherapy-induced alopecia that consider hair volume, hair length, and hair quality is desirable for an appropriate evaluation and generalizability of the efficacy of scalp cooling systems in the future. In turn, this will optimize scalp cooling devices, evaluate the impact on treatment decision-making and patient quality of life, and, as a result, positively promote insurance coverage as a meaningful aspect of cancer supportive care. 

## 5. Conclusions

While chemotherapy is a cornerstone of breast cancer treatment and significantly improves survival rates, it is often associated with adverse dermatologic effects, particularly chemotherapy-induced alopecia. Despite advances in understanding its pathobiology, effective prevention and treatment options remain limited. Scalp cooling is considered oncologically safe and is currently the most promising method for preventing chemotherapy-induced alopecia, with literature supporting its high success rates, particularly in patients on taxane-based, non-anthracycline regimens. It is generally well-tolerated, with no serious adverse events, and is viewed favourably by most patients. However, the heterogeneity of existing research and subjective hair loss assessments limit direct comparisons across studies. The effectiveness of scalp cooling depends on various factors, including the chemotherapy regimen, cooling device, patient characteristics, and cap fit. Achieving and maintaining optimal scalp temperature is critical, and cryogenic cryotherapy may offer potential in this area. Barriers to widespread use include physician awareness and cost. Therefore, further interdisciplinary research and formal guidelines are needed to fully realise scalp cooling’s potential in cancer care.
